# Proceedings of Albany County Medical Society

**Published:** 1874-10

**Authors:** F. C. Curtis

**Affiliations:** Secretary


ART. II.—*Albany County Medical Society. Semi-Monthly Meeting held May 13th,* 1874. Reported by F. C. CURTIS, M. D., Secretary.

Dr. T. D. CROTHERS read a paper "On Impure Milk as a Source of Disease." He said:

Milk as an article of diet is used almost universally. No other nutrient substance is subject to such changes in both its chemical and physiological principles, depending upon various and diversified causes, such as climate, water, food, age, vigor and surroundings. Within a year it has been proved beyond doubt that milk may be, and often is, the active agent or means for the transmission of disease. Late investigations show that the microscopic germs of disease are scattered through milk to a far greater extent than we are aware of. The following case called my attention to this sub-subject. Subsequently I was satisfied that others of similar character have often occurred in this city.

The doctor here detailed a case where he, with other physicians, had treated an entire family for diarrhœa during two months unsuccessfully. A careful inquiry indicated that they used freely the milk from one cow kept in the neighborhood. This cow was found in a close filthy stall, being fed on poor food, and never let out for exercise or fresh air. The milk she gave was poisonous, and in this way it was slowly and surely destroying the health of every person who used it. The family recovered on giving up the use of this milk. Another case was mentioned of an old man recovering from fever, who was ordered to use milk freely as a medicine; a few days after he was seized with diarrhœa and died. Afterwards it was found that the milk came from a poor cow kept in a bad condition, and on bad food, leaving no doubt that the milk was poisonous and the active cause of death. Other cases were related, some of them fatal and others not, but all were found to proceed from impure milk, coming from ill-nourished, half-fed cows. Dr. Crothers discussed at length the three ways in which impure milk may be the source of disease, viz: chemically, physiologically and psychologically.

He remarked that the quality of milk depends upon the good health, exercise, water and vigor of the cow. If any of these elements are wanting, the milk will decrease in proportion. The doctor detailed the experiments of M. Decasne of Paris, drawing the following conclusions: First. A cow produces healthy milk in exact proportion to the surplus of food beyond what is necessary for its own maintenance. If the food is barely sufficient to sustain the normal vigor of the cow, the milk produced must be at a loss of animal tissue, and hence the quality of the milk be deficient and become a prolific source of disease. Second, Insufficient food always causes an imperfect degree of health in the amimal, the extent of which is controlled by the age, condition and surroundings, predisposing to disease and debility, particularly favoring the propagation of disease germs through the milk. Physiologically

(the doctor continued) milk may be normal in quality yet contain some extraneous poisonous matter. He mentioned the epidemics called milk sickness, which had raged through some parts of the West, affecting both animals and man, and evidently communicated by the means of milk. These epidemics came from some poisonous matter taken up as food or water by the cows, causing a dangerous fever, which was transmitted to those who used the milk of these animals. The primal cause was supposed to be a species of fungus eaten by the cow. The doctor also gave details of those startling epidemics of typhoid fever in England and Scotland, which had been traced to the adulteration of milk by poisonous water. In one instance, where thirty-seven out of thirty-nine families supplied by one milkman were attacked with this fever, the disease was traced to the water of a well which received the drainage of a sewer, this water being used to wash out the cans and then thrown into the milk. Here this poisonous water used to adulterate the milk was the source of a fearful epidemic. A similar epidemic occurred near London last year, where over three hundred cases of typhoid fever were traced directly to water containing typhoid fever germs, that had been used in adulterating the milk. Other cases are reported where impure water drank by the cow has caused severe fever among persons who used the milk. Also, a case occurred in this county where cows drank from a well polluted with water coming from a drain, and both the milk and cheese were known to have transmitted the germs of typhoid fever. The milk of cows kept in close, ill-ventilated stables becomes charged with a peculiar stable odor, which is poisonous and the source of many diseases. The doctor mentioned several instances illustrating this, and in conclusion said, these instances show clearly that disease germs of both typhoid and malarious fever, with poisoning from animalculæ and fæcal gases, may be readily propagated through milk.

Psychologically, milk may transmit an unknown influence, coming from the nervous condition of the animal, causing various diseases of a serious nature. Cases of mothers nursing children while excited, causing convulsions and death, were referred to, indicating that milk in such circumstances contained some poisonous element which had a peculiar action on the nervous function. Other instances where epilepsy and chorea had begun in children from nursing when the mother was excited, were also detailed. Dr. Crothers continued: The same disturbing influence from excitement, seen in its disastrous effects in nursing children, may be traced in the milk of cows, only in a less degree. A gentleman in this city bought a young cow to have pure milk for his child. The process of milking was, from ignorance, attended with much excitement and brutality, and the child was seized with brain fever and never entirely recovered—the direct result of feeding this poisonous milk, made so by the excitement of milking. A case was related where a brutal, drunken dairyman supplied four families with milk. Those who used the most milk suffered all the season from gastro-intestinal irritation and a low tone of physical and mental health, due without doubt to the changed and poisonous condition of the milk from excitement. The doctor discussed this subject at length, concluding: First. Milk, to be healthy, must come from well-nourished cows which are having a surplus of food beyond the requirements of the body; also, these cows must have abundance of water, air and exercise. The milk of cows of all ages and conditions is not normal, and may be very unhealthy. Cows kept in stables and fed on grains give impure milk, which is often dangerous. Second. Milk adulterated with water taken from any source, particularly the milk of cows confined in stables, is a very active source and agent of typhoid fever, and other diseases not well known. Third. The milk of one cow, unless the exact conditions of vigor, age and surroundings are known, is not superior to the aggregate milk of a large dairy.

Probably not a single milkman sells pure milk as it comes from the cow. The amount of water varies in good milk, leaving a margin for the milkman which can not be detected by analysis. The milk sold in Albany is not superior to that of other cities. An analysis of ten specimens from as many farms, made by Dr. Tucker of the Medical College, revealed nothing except a large per centage of water. Some of these specimens came from dry upland dairies where springs and wells did not exist, and if the milk had been pure the per cent. should be less; when more, the suspicion of adulteration is strong, and the inquiry should be made, how pure is the water used. Every family using milk largely should know the character and surrounding of the dairy, and the condition of the wells and springs from which water is used by both the dairyman and his cows. We can have no security from this source of danger unless we begin at the first cause. If the milkmen will keep good cows and pure water at all times, comparative immunity from this danger may follow. No subject is of more vital interest to the community, and sanitary neglect here may result disastrously to a great extent. The experience of every day confirms the belief that we have in impure milk a prolific source of diseases now obscure or entirely overlooked.

Dr. STONEHOUSE spoke of a report in the *Edinburgh Medical Journal* of an epidemic of scarlet fever which was traced to the milk from a dairy where persons in the stage of desquamation of this disease had milked the cows, and it was supposed that scales had fallen from their hands into the milk. In another case, milk furnished in London from a certain dairy tasted of creosote, which investigation showed to be due to sprinkling the drain of the milk-room with this article. This circumstance has suggested that the gases from foul drains might be absorbed by milk, and the germs of disease, like typhoid, be conveyed by it.

Dr. L. T. MORRILL reported a case of pelvic cellulitis, resulting in pelvic abscess. Mrs. G., age 39, about eighteen months before her death was confined at full term of a strong, healthy male child. She suffered for a month after with severe pain over the pelvic region. At this time I was called in. Previous to her last confinement she had given birth to nine children, and had four miscarriages. At my first visit I found her complaining of severe pain over the abdomen, which was tympanitic, pulse 110. I diagnosed the case as peritonitis. Two weeks from that time I found her with pulse 90, abdomen slightly tympanitic, accompanied with profuse diarrhœa. At end of the next week peritonitis had almost entirely disappeared, excepting over region of left ovary, and there the pain was intense, and the only relief she could get was from the use of hypodermic injections of morphine. Being naturally of a strong constitution, her general strength held out well. In the course of three or four months she was able to set up, feeling quite well, and recommenced the use of her sewing machine. Every pleasant day she was out, and seemed to gain rapidly.

About this time, when every thing was getting along nicely, her husband came home one night intoxicated, and commenced abusing his wife, pulling her out of bed and kicking her several times over the abdomen. The next morning I found her suffering some pain over the abdomen, which was bruised and swollen; prescribed anodynes and warm fomentations. From that time she suffered extreme pain over lower portion of abdomen, and survived her injuries some eight months. About four months before her death, I saw her late at night, suffering the most extreme pain, delirious, pulse 100. On examining the abdomen I could distinctly feel a tumor about the size of a goose-egg, fluctuating; the slightest touch caused her so much pain that I desisted from further interference that night. I administered one-half grain of morphine hypodermically (she being in the habit of taking large doses of laudanum I was obliged to give her more than the usual amount.) On the following morning I found her in about same condition, but not suffering so much pain. Tumor occupied the same position. On making a vaginal examination, I could feel the tumor projecting to the left of the cervix uteri, or where the cervix ought normally to be—in this case it was thrown to one side, and held there by adhesions. In my manipulations, both internal and external, some pressure was unintentionally brought to bear on the tumor. which gave way, its walls broke, and a discharge commenced and continued to flow from the vagina for four days. I should judge about half a pint in all escaped. Nothing but pus cells could be found on microscopical examination. During and for a short time after the discharge she suffered comparatively little or no pain. The odor from the discharge was extremely unpleasant, and she used a wash of carbolic acid, at the same time she was taking tonics.

For two weeks she seemed to improve, when all at once the pain, heat and swelling returned, and for some twenty days the abscess filled and enlarged, until finally it broke the second time, as previously. During the time of the discharge she suffered no pain to speak of. In like manner the abscess gathered and broke five times, with an intermission of about three weeks, each time discharging more than the time previous. She suffered quite a good deal during the last month from general prostration. Twenty-four hours before her death the abscess broke for the last time, and with the discharge her life seemed to ebb away. She died free from all pain, and perfectly conscious to the last.

A sister of hers, living, is troubled with the same disease. She has had three children and two miscarriages. The tumor forms over region of ovary, same as deceased.

Twelve hours after death a post-mortem examination was made by Dr. Van Derveer—Drs. James Bailey and Bigelow being present. Rigor mortis well marked; body very much emaciated; right lung in a healthy condition, a few slight adhesions, probably of long duration; left lung, adhesions very strong and firm. In the upper portion of the lung was a cavity which would contain about two ounces, empty. Heart small, walls firm, valves normal. Peritoneum and its layers presented some traces of inflammation. In left inguinal region, folds of peritoneum strongly adhered to parietes of pelvis and intestines. Just behind left broad ligament, and under folds of peritoneum surrounding left ovary, were found the walls of an abscess, part of its contents having made its way down back of the uterus, and escaped into the vagina through Douglass' cul de sac; the abscess probably held from ten to twelve ounces. There are two distinct openings into posterior wall of vagina. The fimbriated extremity of the fallopian tube of the left side was firmly included in the peritoneal adhesions. The right broad ligament, appendages and attachments, presented a normal appearance; size of uterus normal; well marked traces of endometritis on laying open the uterus were found. Liver presented appearance of fatty degeneration; no adhesions. Spleen small, firm, and capsule covered with a thick, white fibrinous deposit. Kidneys and other organs in good condition.

Dr. J. S. Bailey mentioned a case somewhat similar, of abscess in the broad ligament, causing peritonitis and death by rupturing. The patient, a stout young woman, and primipara, was delivered twelve days before she died, after a labor presenting nothing unusual in its course, except that it was somewhat protracted, and she complained much of a burning, smarting pain in the right illiac region. She was attended by a midwife, and was only seen in a moribund state. Only a very meagre history could be obtained. An autopsy was made, at which there was found peritonitis, with agglutination of intestines by abundant plastic lymph with more or less pus. An abscess was located in the right broad ligament, which had ruptured into the peritoneal cavity.

Dr. BAILEY also exhibited a biliary calculus of the size of a large butternut, weighing about five drachms. It had completely filled the gall bladder.

---

MAY 27th, 1874.

The Society met at the usual hour and place. The President, Dr. SWINBURNE, in the chair. About twenty-five members present.

After preliminary business was attended to, Dr. WM. H. CRAIG reported a case of *Puerperal Convulsions*, which was treated by veratrum viride and chloral combined, as follows:

Mrs. L., aged 24, primipara, married two years, of feeble constitution, pale and anæmic. Was delivered of a male child weighing 8½ pounds, at 10 o'clock P. M., March 30th, 1874, after an easy, natural labor of ten hours duration. The second stage lasted only two hours, and after taking a nourishing drink, she went to sleep, breathed easy and remained apparently in quiet repose until 2 o'clock, A. M., four hours after delivery, at which time she awoke, complaining of nausea, and while in the act of vomiting, without any other premonitory symptom or exciting cause, (except great nervous excitement for two or three weeks previous), went into a convulsion which lasted about three minutes. She was seen at 2:15 o'clock, soon after which time she had a second convulsion, which was characterized by great muscular contortion, especially of the muscles of the arms, neck and face. The face was livid and there was much frothing at the mouth. When the convulsive action had ceased, the breathing became stertorous with coma, which soon passed off. This was succeeded by unconsciousness and great restlessness, requiring the efforts of two attendants to keep her from getting out of bed. The pulse was 120 per minute and full. She talked inchoherently and complained of headache. On two occasions, previous to marriage, she had had a slight hysterical convulsion, a knowledge of which fact induced me to think hers a case of Hysteria. I gave the bromide of potassium in 20 grain doses every half hour, without producing any modification of the symptoms, the convulsions recurring every fifteen minutes until 4:30 o'clock. I then called Dr. Quackenbush in consultation, who diagnosed it a case of severe puerperal eclampsia. He advised giving her the veratrum viride and hydrate of chloral in combination. Accordingly I sent for Squibb's fluid extract of veratrum, but received instead the officinal tincture. At four o'clock, when the administration of these remedies was begun, the convulsions were occurring regularly every twenty minutes, the pulse 140 and full. Fifteen drops of the tr. veratrum and 20 grains of chloral were given every 15 minutes, and the dose gradually increased until nearly a drachm of the former and 40 grains of the latter were given at a time. At 8 o'clock, A. M., an ounce of the veratrum had been given, yet the pulse remained unchanged, showing the inertness of the officinal tincture. The convulsions were gradually diminishing in frequency, now occurring only once in 40 or 50 minutes, but were very severe.

At 9 o'clock I obtained Burrows' fluid extract of veratrum and gave 20 drops; ten minutes after, the last convulsion occurred. At 10 o'clock I gave 20 drops again and 40 grains of chloral. Pulse was 108.

At 11:30 gave 20 grains chloral and 6 drops of veratrum. 12 o'clock, pulse was 70; veratrum discontinued.

During the afternoon chloral was continued to procure quietness, for as soon as she was allowed to come out from under its influence she became very restless, requiring considerable force to keep her from throwing off the bed clothes and getting out of bed. The pulse rose several times to 90 for a short time, and would again recede to 74. The urine was drawn with the catheter, and on examination, was found to contain a small quantity of albumen, but no casts were found. She had a quiet night, remaining in a sleepy stupor, from which she could be aroused to take nourishment. She would answer questions correctly, but was evidently not altogether conscious. The urine was again drawn with catheter next morning. I gave her one-fourth of a grain of elaterium in two doses, three hours apart, which was followed by free catharsis. After this time no more medicine was administered. She convalesced rapidly, so that at the end of two weeks she was able to sit up more than half the time. Lactation took place naturally, and at this time both mother and child are in the enjoyment of as vigorous health as if they had been the subject of no untoward event. She took, in the space of 18 hours, 280 grains of chloral, 46 drops of the fluid extract of veratrum viride, and one ounce of the officinal tincture of veratrum viride. It is evident that the last is an unreliable preparation in such cases, from the fact that so much was given in the course of four hours without perceptibly affecting the pulse, while the first dose of the fl. ext. caused decided reduction, and in two hours lowered it from 140 to 70 beats in the minute.

The feature which I desire to commend to your attention is the remarkable influence of these two remedies in combination. In the annual address which I had the honor to deliver before this society four years ago, while speaking of the developments yet to be made in therapeutics, I made the following statements: "I believe the next decade will develop many marvelous changes in our knowledge of therapeutics, in which practical medicine will become more demonstrative and less theoretical than heretofore." The case I present to your attention to-night partially verifies the statement then made, for according to my experience and knowledge of the means formerly used in the treatment of similar cases, nothing would have saved this woman's life from a fatal termination. I verily believe she was fairly snatched from the grave by the heroic use of the two substances I have before mentioned.

It also verifies a statement made by Dr. Squibb before the Kings Co. Medical Society, when the use of veratrum in eclampsia was being discussed. He says: "The cases of puerperal convulsions reported here, treated by verat. viride prove to my mind that first there is no fixed dose; the test must be the effect produced, and not the number of minims given. The tendency in therapeutics seems to be to get rid of the trammel of doses." I thoroughly endorse the statement of Dr. Squibb in the above quotation.

Very many cases of puerperal convulsions have been reported in the various medical journals, treated by veratrum alone, and quite successfully; and some fatal cases are mentioned where it seems to me if chloral had been used in combination a disastrous result might have been averted. Other cases have been treated by chloral alone and each remedy has its advocates. I have seen no cases reported where the two have been given in combination.

If we consider the pathology of eclampsia according to the modern theory, which may be expressed in the single word *irritation*, we can then better appreciate the influence of these two remedies in controlling this disease. Dr. Robert Barnes, in a recent lecture relating to the etiology and treatment of puerperal convulsions, gives the following four cardinal principles for our guidance: "1st. To moderate central nervous irritability. 2nd. To cut off emotional irritants or excitants. 3d. To cut off peripheral iritants or excitants. 4th. To eliminate all complicating morbid conditions."

Dr. Radcliff, in a work recently published on disorders of the nervous system, advocates the theory that nervous irritation may be reflected to the nervous centres, through irritation of the uterine nerves, and excitation of the coats of the blood vessels by urea.

Chloral undoubtedly allays the irritation of the nerve centers, while veratrum viride lessens the excitation of the coats of the blood vessels by reducing the vascular action.

The subject of the paper was extensively discussed by members of the Society.

Dr. QUACKENBUSH remarked that he had little to add to what Dr. Craig had said. In regard to the cause of puerperal convulsions, this is a point not well settled. Albuminuria usually exists with their occurrence, but why this renal affection should occur is a question. Simple pressure of the enlarged uterus cannot alone produce it, for there is much greater enlargement with ovarian and fibroid tumors, without its occurrence. Barnes thinks it is due to overtaxing the kidneys in eliminating effete matters. Braxton Hicks believes this is the case, and also that this same poisonous principle may affect the brain directly.

The pathological condition is thought by many to be only a congestion of the brain; by others to be simply an excitement of the nervous system.

The treatment has been to cause rapid delivery, if not complete, and quiet the great nervous excitability. For the latter chloroform has generally been used. Chloral acts much in the same way, but is more permanent in its effects. The object of combining veratrum viride with this is to combat the rapid action of the heart. By doing this the force of the pulsation is relieved, and too, supposing a poisonous principle to be in the blood, causing the convulsions, the quantity of this carried to the brain is diminished.

Dr. J. S. BAILEY mentioned a case which had been reported in one of the journals where 250 grains of chloral were given in a few hours, 15 grains every 15 minutes, without retarding labor. He also spoke of giving this drug by enema as recommended by Dr. Gross in a recent discussion on chloral in Philadelphia.

The PRESIDENT remarked that in the treatment of this disease with chloroform by inhalation, a majority of the patients have recovered. He had kept patients under its influence for 24 hours at a time. He doubted whether better results were to be obtained by other means. He had little faith in veratrum in its general use.

Dr. HALE spoke of a case under his care in which the patient, who had at her previous confinment convulsions for which chloroform had been given, was in the one at which he attended her very restless, excitable and sleepless. Labor pains came on and continued every day for four weeks, during which time she had headache, often quite severe. To control this and avert the possible recurrence of the convulsions, chloral and chloroform were given, in all during the time, but most of it toward the last, 350 grains of the former and 10 ozs of the latter. The day before delivery, neither of these controlling the restlessness, $\frac{1}{8}$ grain of morphine was given. She passed into a comatose state after the birth of the child, and died next day.

*Post mortem* the stomach was found very soft, tearing in lifting it out. It was ruptured, as was also the diaphragm, and about 12

ozs of blood was in the left pleura. There was a very decided smell of chloral about the stomach and its contents.

Dr. SWINBURNE did not think we could attribute this softened condition of the stomach to the action of chloral. It is a condition not frequently met with apart from exciting cause. He mentioned a case where it was found in a case of sudden death; the stomach was examined by Dr. Alonzo Clark and others, who declared that it was softened by the gastric juices.

Dr. STONEHOUSE said that at Sandford Hall chloral was used a good deal, 40 grains a day for long periods being constantly given. It was not noticed to produce stomach derangement; on the contrary, it was thought to be beneficial in dyspepsia.

Drs. JONES and BAILEY spoke on the use of chloroform by the stomach, finding it more prompt in action and certain in its effects.

Dr. QUACKENBUSH thought there were cases of puerperal convulsions when bleeding is applicable. We are floating toward the theory that all these cases are due to uremic poisoning. It is better to take the view that it may be due to inflammation, and in suitable cases bleeding will relieve more certainly than anything else.

Dr. F. C. CURTIS reported a case of *Tubercular Ulceration of the Larynx.* The patient, a young man, aged 28, was first seen three months before he died. He complained of sore throat and hoarseness, which he had been troubled with for a year. He could not speak above a whisper. This he referred to immoderate use of his voice in driving cattle and general lack of care of himself. Examination with the Laryngoscope showed an extensive ulcer on the right side of the larynx posteriorly, in front of the arytenoid cartilage, and just above the vocal cord, the mucous membrane of which only was affected. There was considerable swelling of the posterior portion of the larynx, and a general erythematous condition of the entire part, though no active inflammation. The fact was further elicited that he had coughed for several years at times; more of late. Examination of lungs showed consolidation of both apices. It was only quite recently that he had begun to fail so as to be obliged to quit work. His family history was clear of phthisis.

Treatment was directed solely to his general condition, and little attention was paid the throat, the symptoms of which did not increase in gravity. The lung symptoms did however. Softening began and proceeded rapidly. He died three months after.

*Post mortem* examination showed the lungs very extensively affected. In fact only the thin edges of the lobes crepitated naturally. The lower lobes were filled with milliary nodules; higher they were the size of a hazelnut and larger, while at each apex were large, ragged cavities.

In the larynx there were found two ulcers, one as already described on the right side; the other directly opposite, in the ventricle, being merely a chink, itself hardly visible in the natural condition of the parts, but with considerable superficial ulceration about it. Superior to these ulcers there was œdema of the tissues. The epiglottis and anterior portion of the larynx was not affected except by congestion.

In regard to the pathology of these ulcers occurring with pulmonary consumption, authorities are divided as to their being due to actual deposit locally of tubercle. However, Virchow and most of the best pathologists believe in the posibility of this, and that a portion of them are so induced. Otherwise they are due to inflammation and breaking down of mucous membrane, glands or follicles, etc.

As to diagnosis the ulcer itself has litt e peculiar to itself. Location of the ulcer is most important; as a rule they affect the posterior portion of the larynx, while those of syphilis and cancer may be looked for on the anterior. Cancer is usually painful. Aphonia more commonly occurs with tubercular ulcers.

Diagnosis is important, as the treatment turns much upon it. These ulcers do not bear active treatment, and it was believed should be soothed instead. Inhalation of carbolic acid is recommended, the local application of morphine if indicated, and the like. The general condition which they accompany will, as a matter of course, be looked after.

On account of the lateness of the hour there was no discussion of the paper.

On motion of Dr. Stonehouse, a committee of five was appointed to prepare a memorial to the Board of Supervisors in reference to the erection of an asylum for the insane in this county.

The Society then adjourned.

---

*Semi-Annual Meeting,*
JUNE 9th, 1874.

The Society met at the City Hall, and in the absence of the president, was called to order by Dr. W. H. BAILEY, who was elected president *pro tem.*

After some matters of business were disposed of, the question of urging the erection of a county insane asylum was discussed. A resolution was adopted, expressive of satisfaction that the Board of Supervisors and Aldermen had the matter already under consideration, and heartily endorsing the object.

The secretary presented the act recently become a law, "to regulate the practice of medicine and surgery in the State of New York." It was referred, without discussion, to the Board of Censors.

Dr. BOYD, chairman of the Board of Censors, reported the following names, and recommended their election to membership on complying with the by-laws:

Drs. L. Balch, O. D. Ball, R. F. Barton, G. H. Benjamin, L. Bondrias De Morat, C. E. Buffinton, D. H. Cook, H. C. Everts, Wm. Geoghegan, Jr., C. S. Merrill, N. Monroe, L. T. Morrill, G. W. Papen, F. Weidman, W. M. McGregor, G. L. Van Allen, A. T. Van Vranken, J. A. Hart and J. L. Archambeault.

The report was adopted. Matters of minor importance were attended to, and the Society adjourned.

